# Single port/incision laparoscopic surgery compared with standard three-port laparoscopic surgery for appendicectomy: a randomized controlled trial

**DOI:** 10.1007/s00464-014-3416-y

**Published:** 2014-10-01

**Authors:** The SCARLESS Study Group

**Affiliations:** Aberdeen, Scotland

**Keywords:** Single port/incision laparoscopic surgery, Randomized controlled trial, Appendicectomy, Cosmesis, Pain

## Abstract

**Background and objective:**

The aim of this study was to compare the effectiveness of single port/incision laparoscopic surgery (SPILS) with standard three-port laparoscopic surgery for appendicectomy in adults. Feasibility data was collected to evaluate generalizability to other single-port techniques such as cholecystectomy.

**Methods:**

This was a single-center, randomized controlled trial. Participants were randomized to receive either SPILS or standard three-port laparoscopic appendicectomy. The primary patient-reported outcomes were body image and cosmesis at 6 weeks. The primary clinical outcome was pain at 1–7 days. Secondary outcomes included duration of operation, conversion rates, complication rates, use of analgesia, hospital re-admission rates, re-operation rates, and time to return to normal activities.

**Results:**

Seventy-nine patients were randomized. Sixty-seven completed the day 1–7 diary and 53 completed the 6-week follow-up. SPILS patients answered significantly more favorably to the items in the body image scale [mean (SD) 5.6 (1.0) vs. 7.0 (3.3); −1.4 (95 % CI −2.8 to 1.5; *p* *=* 0.03)] and the cosmetic scale [18.9 (4.1) vs. 15.3 (5.8); 3.6 (95 % CI 0.7–6.5; *p* *=* 0.016)] compared with patients in the Standard group. The duration of operation was shorter for SPILS, and patients required less morphine in recovery; however, there were no statistically significant differences in other outcomes.

**Conclusions:**

Patient-reported body image and cosmesis outcomes were better, and surgical outcomes were similar following SPILS. However, the SPILS procedure is more technically demanding and may not be achievable or necessary in routine clinical care. Further assessment of the findings is needed through larger multicenter studies.

Laparoscopic surgery is the preferred approach for many abdominal procedures because of reduced postoperative pain, more rapid recovery, and improved cosmesis which follows a successful operation compared with a conventional single large incision. Whilst the long-term clinical result may be similar [1], the perception amongst many patients and surgeons of the advantage in terms of these short-term outcomes is a powerful influence on practice. There are continuing developments to laparoscopic surgery to reduce the size, number, and placement of incisions to both improve the cosmetic appearance and reduce abdominal wall trauma.

One of the recent innovations is single port/incision laparoscopic surgery (SPILS). This can be either insertion of multiple ports through a small incision or through a proprietary device with multiple channels. The fundamental difference to conventional multi-port laparoscopic surgery is to place all the ports through a single incision which, when sited in the umbilicus, can result in no visible scar in the abdominal wall.

The current literature largely comprises case reports and small series detailing single-port methods. The technique has been used to perform a large variety of procedures, including appendicectomy, cholecystectomy, nephrectomy, hysterectomy, oophorectomy, adrenalectomy, gastric bypass, Nissen fundoplication, hernia repair, splenectomy, colon resection, and liver resection. Apart from a handful of reported randomized controlled trials (RCTs), [2–6], the evidence base is insufficient to inform practice and robustly assess claims of reduced pain and morbidity with improved cosmesis and faster recovery [7–12]. In general, it is perceived that the single port/incision technique takes longer than conventional laparoscopic surgery and the differences in costs and safety are unknown.

Nevertheless, there has been considerable interest in introducing single port/incision surgery, and a large number of training courses are available. The public perception is that it might become the procedure of choice if it becomes widely available [13]. It is crucial that the technique be critically evaluated during the introductory phase of implementation to provide robust data to inform further adoption and evaluation. However, the difficulty of undertaking such an evaluation has been succinctly stated in Buxton’s law: “It is always too early (for rigorous evaluation) until, unfortunately, it’s suddenly too late” [14]. Ideally, a definitive evaluation requires a large, multicenter RCT. Despite the recent publication of clinical trials in appendicitis [15, 16] and observational studies [17], there remains a paucity of data to help plan and design a large RCT or justify widespread adoption of SPILS. Additionally, further refinement of the single port/incision technique is needed.

The aim of this study was to compare the effectiveness of single port/incision laparoscopic appendicectomy with standard three-port laparoscopic appendicectomy in adult patients at 6 weeks post-surgery. Appendicectomy was the focus of this study because it is a common and relatively simple procedure to undertake. Feasibility data were collected to evaluate generalizability to other more complex single-port techniques such as cholecystectomy.

## Material and methods

Ethical approval was given by the North of Scotland Research Ethics Committee (REC reference number 10/S0802/77). Adult patients aged over 16 years presenting with suspected appendicitis for whom laparoscopic surgical management was judged appropriate were eligible for the trial. Patients were identified in the General Surgery Units, Aberdeen Royal Infirmary (UK), by the consultant or designated team member. Patients who had previous open abdominal surgery through midline incision or previous umbilical hernia repair with mesh were excluded. Participants were randomized to receive SPILS or standard surgery in equal proportion using the randomization application at the trial office at the Centre for Healthcare and Randomised Trials (CHaRT), Aberdeen. Randomization was by computer-generated permuted blocks of size two and four and stratified by gender. Because of the acute nature of the admission to surgery and potential difficulty in tracking patients, date of birth was also recorded and used in addition to the study number to identify patients.

The surgical interventions were delivered or supervised by a surgeon who had expertise in the specific intervention. A standard anesthetic regimen and pain-relief policy was followed, where possible. Ports sites of 10 mm and over were closed with absorbable sutures before closing the skin. The two interventions being compared were:

1. *Single-port laparoscopic (SPILS) surgery* A single intra-umbilical incision was made and a multi-channel port inserted. A 5 mm, 30 degree telescope was used to visualize the operative field. Conventional laparoscopic instruments were used for the procedure. Roticulating/curved instruments were available and used if required. Use of any additional instruments or ports was recorded for cost analysis.
2. *Standard three-port laparoscopic surgery* Pneumoperitoneum was established by an open technique through an intra/supraumbilical incision with a 10–12 mm port for initial pneumoperitoneum and inspection. A further 5 or 10 mm port was used in the left iliac fossa (depending on the availability of 5 mm laparoscopes) and a 5 mm port inserted in the hypogastrium. Standard laparoscopic instruments were used for the procedure as per existing hospital protocol.

### Outcomes

The primary patient-reported outcome (PRO) measure was patient-reported cosmesis and body image using the Body Image Questionnaire (BIQ) [18] at 6 weeks. The primary clinical outcome was severity of pain using the pain Numerical Rating Scale (NRS) at 1–7 days. Other PROs included the Hospital Experience Questionnaire (HEQ) [18], analgesic usage, and time to return to normal activities. Other clinical outcomes included analgesic use, duration of operation (minutes) and complication rates, conversion rates, infection rates (intra-abdominal and wound), related hospital re-admission rates up to 6 weeks, re-operation rates and port-site hernia up to 6 weeks. In addition, the following feasibility measures were collected: eligible patients per month, proportion formally considered for trial entry, proportion randomized (and reasons why not), proportion who were unaware of their received intervention at 24 h, proportion of those recruited with a complete data set at 6 weeks, surgeon’s perception of SPILS approach and the suitability of available equipment. Resource-use data was limited to operative time, theatre time, and length of stay.

### Data collection and processing

Participants were assessed pre-operatively to confirm eligibility, and perioperative data was collected. PROs were collected by diary completed on days 1–7 following surgery and by postal questionnaires at 6 weeks post surgery. Patients were not reviewed post-discharge unless there was a clinical indication following current clinical practice.

### Sample size

As there were no published RCTs upon which to base it, a formal sample size calculation based upon previous data was not possible. A sample of 80 participants recruited over the 7-month recruitment phase was anticipated. Adopting a 5 % two-sided significance level, this would allow an effect size (Cohen’s d) of 0.65 to be detected with 80 % power for patient-reported measures such as the BIQ [equivalent to mean difference of 1.8 based upon standard deviation (SD) of 2.8].

### Statistical analyses

Data was described using number and percentage, mean and SD, or median and interquartile range (IQR) as appropriate. All estimates of intervention are presented with 95 % confidence intervals (CIs). Dichotomous variables were analysed using Fisher’s exact test and 95 % CIs derived using Newcombe’s method [19]. Categorical data were analysed using a *χ* ^2^ test for trend [20]. Continuous data were analysed using *t* tests. Pain data from day 1 to 7 were converted to an area under the curve (AUC) and reported and analysed as a continuous outcome, and summarized graphically. For feasibility measures, such as the proportion of eligible patients who consent to randomization, the frequency was calculated. All analyses were by intention-to-treat on complete cases only and software used was Stata 12 [21].

## Results

**Fig. 1:**
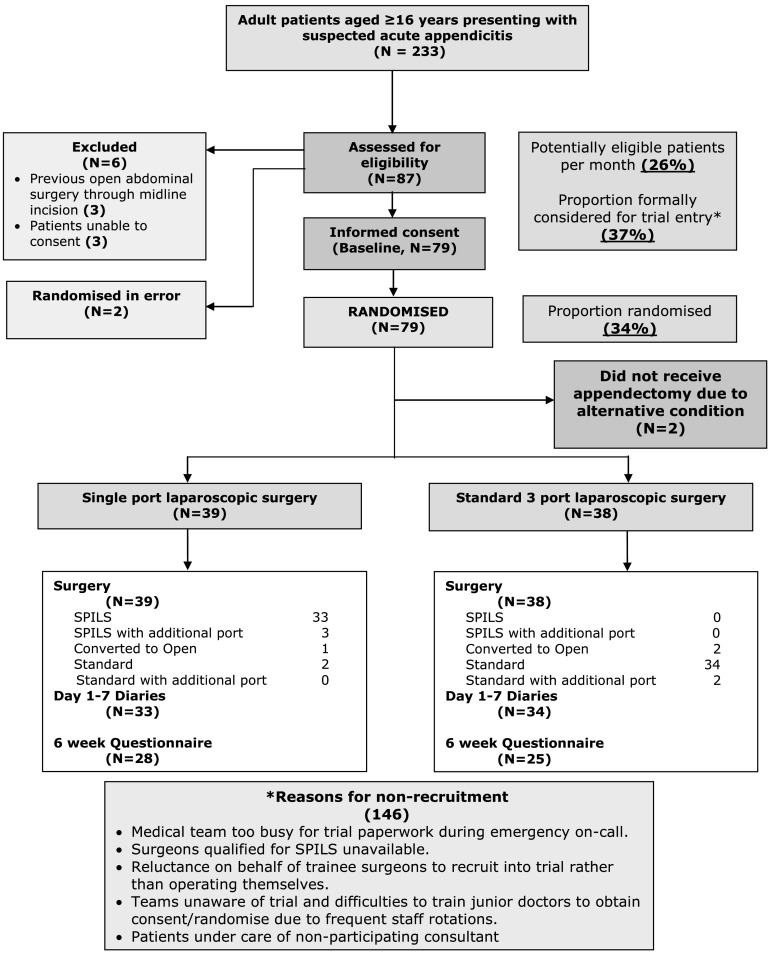
Consort diagram/flowchart. *SPILS* single port/incision laparoscopic surgery

Recruitment began on 8 January 2011 and concluded on 16 September 2011. A total of 233 adult patients aged 16 years or over presented with suspected acute appendicitis. Eighty-seven patients were formally approached; of these, three patients were ineligible due to previous open abdominal surgery through midline incision and three patients were unable to consent. Seventy-nine patients were eligible and agreed to take part in the study, and were randomized to SCARLESS (see CONSORT diagram, Fig. 1); 39 patients were allocated to the SPILS group and 40 patients were allocated to the Standard group. Two patients in the Standard group underwent surgery other than appendicectomy (one patient underwent surgery for a perforated sigmoid colon; one patient underwent a cholecystectomy) and therefore were regarded as post-randomization exclusions and were not included in the statistical analyses. Baseline characteristics were generally well balanced in terms of age, gender, American Society of Anesthesiologists (ASA) grade and body mass index (BMI) (Table 1).

**Table 1:** Baseline characteristics

	SPILS (*n* *=* 39)	Standard (*n* *=* 38)
Female [*n* (%)]	19 (49)	18 (47)
ASA grade [*n* (%)]		
1	29 (74)	32 (84)
2	3 (8)	1 (3)
3	1 (3)	
Missing	6 (15)	5 (13)
Age [years; median (IQR)]	27 (19–45)	32 (21–38)
BMI [median (IQR)]	25 (22–29)	26 (23–29)

*SPILS* single port/incision laparoscopic surgery, *IQR* interquartile range, *BMI* body mass index

For the majority of patients in both groups, the main surgery was undertaken by a junior or senior trainee surgeon—31 (79 %) and 35 (92 %) for the SPILS and Standard groups, respectively; a consultant surgeon was present in five (13 %) of the SPILS operations and three (8 %) of the Standard operations. Only three patients in the SPILS group were operated on by a consultant surgeon (Table 2). The vast majority of patients in both groups received their allocated intervention—33 (85 %) and 34 (89 %) for the SPILS and Standard groups, respectively. Three patients in the SPILS group required an additional port, two patients underwent standard three-port laparoscopic surgery, and one patient was converted to an open operation. In the Standard group, two patients required an additional port and two patients were converted to an open operation.

**Table 2:** Operative details

	SPILS (*n* *=* 39)	Standard (*n* *=* 38)
Duration of operation		
Total time (mins)		
Mean (SD)	74 (23)	89 (37)
Median (IQR)	70 (60–90)	80 (70–100)
Surgery time (mins)		
Mean (SD)	48 (20)	62 (26)
Median (IQR)	40 (35–60)	58 (45–70)
Medication during anesthesia [*n* (%)]		
Bupivacaine adrenaline	33 (85)	34 (89)
Suxamethonium	31 (79)	25 (66)
Morphine	37 (95)	37 (97)
Fentanyl	35 (90)	31 (82)
Surgery received [*n* (%)]		
Received allocated intervention	33 (85)	34 (89)
Received allocated intervention with additional port	3 (8)	2 (5)
Received standard three-port surgery	2 (5)	NA
Conversion to open surgery	1 (3)	2 (5)
Surgeon [*n* (%)]		
Consultant surgeon	3 (8)	0 (0)
Junior/senior trainee with consultant	5 (13)	3 (8)
Junior/senior trainee	31 (79)	35 (92)
Surgeon-rated difficulty of operation [*n* (%)]		
Straightforward	15 (38)	20 (53)
Mildly difficult	7 (18)	7 (18)
Moderately difficult	11 (28)	9 (24)
Extremely difficult	6 (15)	2 (5)

*SPILS* single port/incision laparoscopic surgery, *SD* standard deviation, *IQR* interquartile range, *NA* not applicable

There were four cases of intraoperative complications in three patients allocated to the Standard group—two cases of bleeding, and two reported injuries to the abdominal viscera. Intraoperative complications have been summarized in Table 3. There were few cases of postoperative complications in both groups, with three (8 %) patients in the SPILS group experiencing at least one complication compared with five (13 %) in the Standard group. The most common postoperative complication was surgical site infection, with two (5 %) and three (8 %) cases for the SPILS and Standard groups, respectively. Three participants required a re-operation: in one patient with a retrocaecal-subhepatic appendix abscess undergoing SPILS, despite the insertion of a supplementary port the appendix was thought to have been excised but a second operation was required 3 days later to remove the appendix; one patient in the Standard group required a re-operation due to bowel injury and the other had a re-operation for a pelvic abscess. These complications and re-operations are summarized in Table 3.

**Table 3:** Surgical complications and re-operations

	SPILS (*n* *=* 39) [*n* (%)]	Standard (*n* *=* 38) [*n* (%)]	Difference	95 % CI	*p*-value
Intraoperative complications					
Bleeding	0	2 (5)	−5	(−17 to 5)	0.24
Injury to abdominal viscus	0	2 (5)	−5	(−17 to 5)	0.24
Postoperative complications					
Surgical site infection	2 (5)	3 (8)	−3	(−16 to 10)	0.67
Äbdominal viscus injury	0	1 (3)	−3	(−14 to 16)	0.49
Respiratory infection	0	1 (3)	−3	(−14 to 16)	0.49
Chest pain	1 (3)	0	3	(−7 to 13)	1
Re-operations					
Failure to identify appendix	1 (3)	0	3	(−7 to 13)	1
Bowel injury	0	1 (3)	−3	(−13 to 7)	1
Pelvic abscess	0	1 (3)	−3	(−13 to 7)	1

95 % CI estimated using Newcombe’s method; *p*-value from Fisher’s exact test

*SPILS* single port/incision laparoscopic surgery

On average, SPILS was quicker than standard surgery, with the total operation time being 15 min shorter (95 % CI 0–28; *p* = 0.048; Table 2). Other resource-use outcomes are detailed in Table 4. The hospital re-admission rate was higher in the Standard group. Length of hospital stay was similar in both groups, as was return to normal activities.

**Table 4:** Resource usage and time to normal activities

	SPILS (*n* *=* 39)	Standard (*n* *=* 38)	Difference	95 % CI	*p*-value
Length of stay [days]					
Mean (SD)	3 (2.8)	2.8 (4.4)			
Median (IQR)	2 (1–3)	1 (1–3)			
Hospital re-admission [*n* (%)]	2 (5)	7 (18)			

	SPILS (*n* *=* 28)	Standard (*n* *=* 25)			
Returned to normal activities at 6-weeks [*n* (%)]	24 (86)	19 (76)	10	(−12 to 31)	0.49

*Only participants who are in paid employment:*					
	SPILS (*n* *=* 17)	Standard (*n* *=* 17)			
Time to normal activities (days)					
Mean (SD)	15 (11)	20 (15)			
Median (IQR)	12 (9–22)	14 (8–35)			

95 % CI estimated using Newcombe’s method; *p*-value from Fisher’s exact test

*SPILS* single port/incision laparoscopic surgery, *SD* standard deviation, *IQR* interquartile range

**Fig. 2:**
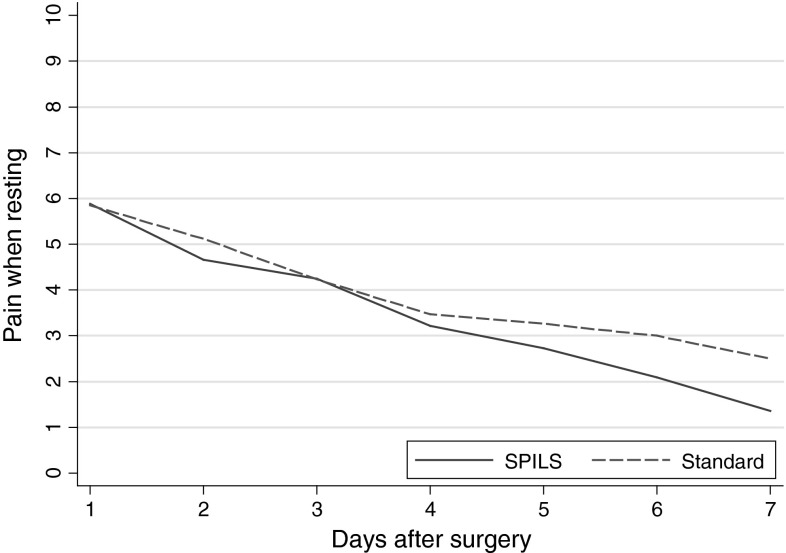
Self-reported pain whilst resting from 7-day diary. *SPILS* single port/incision laparoscopic surgery

**Fig. 3:**
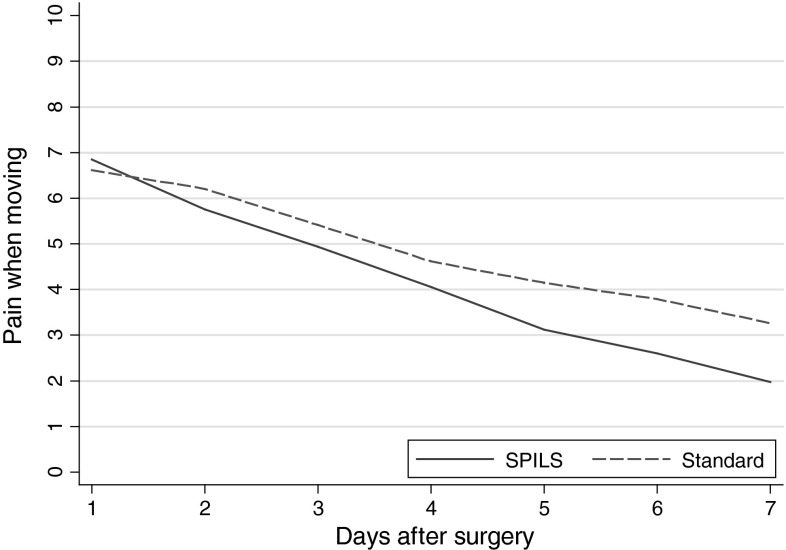
Self-reported pain whilst moving from 7-day diary. *SPILS* single port/incision laparoscopic surgery

Morphine use during immediate recovery was less in participants in the SPILS group than in the Standard group—16 (41 %) versus 29 (76 %), respectively [difference −35 % (95 % CI −53 to −13); *p* *=* 0.003]. Morphine dose received was similar in both groups when given. During immediate recovery, 26 (67 %) participants in the SPILS group versus 32 (84 %) in the Standard group required paracetamol—17 % less (95 % CI −2 to 35; *p* *=* 0.097). There were no differences in the use of postoperative analgesia on the ward. Similarly, there was no statistical difference in patient-reported pain on days 1–7 (Table 5; Figs. 2, 3).

**Table 5:** Postoperative pain and use of analgesia

	SPILS (*n* *=* 39) [*n* (%)]	Standard (*n* *=* 38) [*n* (%)]	Difference	95% CI	*p*-value
Post-operative analgesia in recovery room					
Paracetamol	26 (67)	32 (84)	−17	(−35 to 2)	0.097
Morphine	16 (41)	29 (76)	−35	(−53 to −13)	0.003
Morphine dose (mg)					
All participants					
Mean (SD)	2.8 (4.3)	5.6 (4.3)			
Median (IQR)	0 (0–4)	6 (2–10)			
Only participants who received morphine (excluding those who did not receive any)					
Mean (SD)	6.8 (4.2)	7.4 (3.3)			
Median (IQR)	5 (4–11)	8 (4–10)			
Post-operative analgesia on ward					
Any use of					
Paracetemol	35 (90)	30 (79)	11	(−6 to 27)	0.22
Dihydrocodeine	20 (51)	16 (42)	9	(−13 to 30)	0.50
Diclofenac	5 (13)	6 (16)	−3	(−19 to 13)	0.76
Morphine	7 (18)	12 (32)	−14	(−32 to 6)	0.19

	SPILS (*n* *=* 33) [AUC mean (SD)]	Standard (*n* *=* 34) [AUC mean (SD)]			
Patient-reported pain during days 1–7 post-operation					
Pain when resting^a^	19.4 (11.9)	22.4 (10.8)	−3	(−9.6 to 3.5)	0.36
Pain when moving^a^	23.5 (11.9)	29.2 (12.2)	−5.7	(−12.7 to 1.2)	0.10

95 % CI estimated using Newcombe’s method; *p*-values from Fisher’s exact test unless denoted

^a^Differences, 95 % CI and *p*-value from *t* test

*SPILS* single port/incision laparoscopic surgery, *SD* standard deviation, *IQR* interquartile range, *AUC* area under the curve

Twenty-one (75 %) patients in the SPILS group compared with 14 (56 %) patients in the Standard group considered their stay in hospital to be ‘not too long, not too short’. However, eight (32 %) patients in the Standard group thought that their stay was ‘a little bit too short’ compared with three (11 %) in the SPILS group. The difference between groups was not statistically significant. Overall, the majority of patients in both groups indicated that their treatment in hospital was ‘good’ or ‘very good’—27 (96 %) in the SPILS group compared with 19 (76 %) in the Standard group (*p* *=* 0.012). There were no statistically significant differences in pain after the operation or length of time taken to eat normally (reported at 6 weeks). The HEQ has been summarized in Table 6.

**Table 6:** Hospital Experience Questionnaire

	SPILS (*n* *=* 28)	Standard (*n* *=* 25)	*p*-value
Admission experience [*n* (%)]			
A little bit too long	2 (7)	2 (8)	0.17
Not too long, not too short	21 (75)	14 (56)	
A little bit too short	3 (11)	8 (32)	
Much too short	1 (4)	1 (4)	
Missing	1 (4)	0 (0)	
Treatment [*n* (%)]			
Bad	0 (0)	2 (8)	0.012
Reasonable	1 (4)	4 (16)	
Good	12 (43)	12 (48)	
Very good	15 (54)	7 (28)	
Pain after operation [*n* (%)]			
No pain at all	1 (4)	1 (4)	0.43
A little bit of pain	9 (32)	7 (28)	
Quite a lot of pain	13 (46)	9 (36)	
A lot of pain	5 (18)	8 (32)	
Long time to eat normally [*n* (%)]			
No, not at all	8 (29)	10 (40)	0.99
Yes, a little time	15 (54)	8 (32)	
Yes, quite a lot of time	4 (14)	3 (12)	
Yes, extremely long	1 (4)	4 (16)	

*p*-value from *χ* ^2^ test for trend

*SPILS* single port/incision laparoscopic surgery

At 6 weeks’ follow-up, patients in the SPILS group answered the items in the BIQ significantly more favorably: body image score [mean (SD)] 5.6 (1.0) versus 7.0 (3.3) for the SPILS and Standard groups, respectively; difference in means −1.4 (95 % CI −2.8 to 1.5; *p* *=* 0.03). The cosmetic score was also answered significantly more favorably in the SPILS group; mean difference 3.6 (95 % CI 0.7–6.5; *p* *=* 0.016). However, there were no differences in postoperative self-confidence [7.9 (1.8) vs. 7.1 (2.7); see Table 7].

**Table 7:** Patient-reported pain, body image, and cosmesis

	SPILS (*n* *=* 27) [mean (SD)]	Standard (*n* *=* 25) [mean (SD)]	Difference	95 % CI	*p*-value
Patient-reported pain at 6 weeks					
Pain when moving	0.3 (0.5)	0.4 (0.8)	−0.1	−0.5 to 0.2	0.46
Pain when resting	0.4 (0.7)	0.6 (1)	−0.2	−0.7 to 0.3	0.47
Body Image Questionnaire					
Body image scale	5.6 (1.0)	7.0 (3.3)	−1.4	−2.8 to 1.5	0.03
Cosmetic scale	18.9 (4.1)	15.3 (5.8)	3.6	0.7 to 6.5	0.016
Confident after	7.9 (1.8)	7.1 (2.7)	0.7	−0.5 to 2.0	0.24

95 % CI and *p*-value from *t* test

*SPILS* single port/incision laparoscopic surgery, *SD* standard deviation

No statistically significant differences in self-reported pain were detected between groups at 6 weeks: mean (SD) pain when resting 0.3 (0.5) in the SPILS group versus 0.4 (0.8) in the Standard group, difference in means −0.1 (95 % CI −0.5 to 0.2; *p* *=* 0.46); pain when moving 0.4 (0.7) versus 0.6 (1), difference in means −0.2 (95 % CI −0.7 to 0.3; *p* *=* 0.47) [Table 7].

### Feasibility measures

The proportion of potentially eligible patients recruited to the study was 79/233 (34 %), which represented a rate of approximately eight per month (Fig. 1). A substantial proportion of patients were not approached or assessed for eligibility due to the emergency setting of treatment in this study. Reasons included lack of awareness of the study amongst surgical teams due to frequent staff rotations, reluctance of trainee surgeons to recruit due to perceived threat to surgical training, reluctance of consultants to participate because of an unknown safety profile or due to time pressure, and non-availability of a surgeon with expertise in SPILS. It was intended to train all participating surgeons in both techniques but in the event this proved impractical and the majority of SPILS procedures were performed by senior surgical trainees, whereas the standard three-port surgery was performed by surgeons at differing levels of expertise. Only 15 (38 %) surgeons in the SPILS group rated the operation as straightforward compared with 20 (53 %) in the Standard group, with more surgeons rating the operation as moderately difficult [11 (28 %)], or extremely difficult [6 (15 %)] compared with 9 (24 %) and 2 (5 %) for Standard three-port surgery (Table 2). Blinding of participants to the allocation by the use of bandages was abandoned after seven participants due to the difficulties in preventing details of the surgery being communicated to the patients by medical staff not directly involved in the study. As a consequence, the proportion unaware of their received intervention at 24 h was not available. The number of completed 6-week questionnaires and full datasets were 53 (67 %) and 51 (65 %) respectively. Apart from the availability of a multichannel port and 5 mm laparoscope no additional equipment was required to deliver SPILS for appendicectomy.

## Discussion

The SCARLESS study has shown that patient-reported pain and resource-use outcomes were similar, and patient-reported body image possibly better, following SPILS when compared with Standard three-port laparoscopic appendicectomy. This was achieved in an emergency setting in a single teaching hospital where special arrangements for surgical cover had to be adopted, due to the innovative nature of SPILS, to facilitate the conduct of the trial. Such arrangements are unlikely to reflect actual care if SPILS were to be routinely adopted in preference to a Standard three-port laparoscopic procedure. The reduced surgical access and visualization of a single-port approach may compromise patient safety, as in the case where the appendix was not removed and a second operation was required. Therefore, further assessment of the relative merits of the procedures is needed through larger multicenter studies. However, there are a number of practical challenges to conducting such studies in a way that provides generalizable findings.

In SCARLESS, the SPILS procedure was carried out by surgeons with expertise in the conventional three-port laparoscopic procedure and had been trained to undertake SPILS. The shorter operating time observed in SCARLESS may be due to more experienced surgeons in the SPILS group or due to the lack of time and opportunity to extend SPILS training to all the junior staff. Due to the nature of appendicectomy, as one of the general surgical operations that form part of surgical training, the greater number of surgeons with a wider range of experience who delivered the three-port procedure limits the generalizability of the study findings. Successful completion of the operation was similar, although more cases required modification of the initial approach following SPILS, whether through use of an additional port or conversion to open surgery.

Patient-reported pain was similar between groups. There appeared to be slightly less pain following SPILS; however, this was not statistically significant. The SPILS group required less morphine in recovery with a lower, although not statistically significant, intensity post-operative analgesia following initial recovery prior to discharge. Body image data favored SPILS, with a statistically significant difference for two of three outcomes, although it is uncertain how important such differences are to patients. A general favoring of SPILS from a patient perspective is not surprising if other outcomes were at least as good. This was observed in a recent survey of patient preferences [22].

A recently published systematic review identified a number of studies that had assessed single-port appendicectomy; however, the vast majority were uncontrolled (case series) studies [17]. Two RCTs have been published comprising 40 patients [15] and 360 patients [16]. In general, these showed similar outcomes for the two procedures, although, in contrast to SCARLESS, both had a shorter operation time for the three-port procedure. However, the differences in operation time for all RCTs, including SCARLESS, were not of a magnitude that would clinically have much consequence. A handful of observational studies also reported similar findings [17].

Recruitment was relatively fast and in keeping with expectation. However, this masked the practical challenges of conducting a randomized trial in an emergency setting where multiple surgical teams utilize the same facilities in a fluid process. The acute nature of the admission to surgery made it difficult to track and consent potential participants. A proportion of cases required treatment out with regular hours. Another problem related to the reluctance of some surgical staff to pass on information about potential eligibility due to concerns over safety and the perceived threat to their surgical training. In addition to the study’s surgical investigators, a part-time research nurse was involved in the recruitment process, which was highly beneficial. However, without full-time dedicated recruitment staff, improved rate of assessment for recruitment would be unlikely in the hectic context of an acute surgical unit. Subsequent in-patient follow-up of participants was also difficult, some moving wards multiple times; this was reflected in the completeness of the dataset. Return of the 6-week questionnaire was also relatively low despite the use of reminders, possibly reflecting the demographics of the participants, who were a young and active group.

We abandoned our attempt to blind participants part way through the study due to impracticality, both in applying three dressings to all patients and in preventing knowledge of surgery being communicated by medical staff not directly involved in the study. As such, participant perceptions, possibly influenced by medical staff, could have influenced patient-reported pain. Nevertheless, the findings are consistent with intuitive expectation that, other things being equal, participants would prefer SPILS if surgical outcomes were at least similar. Furthermore, the usage of morphine was lower following SPILS, which could be seen as a ‘stronger’ sign of a genuine reduction in pain.

## Appendix

### SCARLESS Group (in alphabetical order)

Mr Irfan Ahmed, Department of Surgery, Aberdeen Royal Infirmary; Dr Jennifer Burr, Mrs Mayret Castillo, Ms Diane Collins, Dr Jonathan A. Cook, Professor Marion Campbell, Dr Anne Duncan, Health Services Research Unit, University of Aberdeen; Mrs Anu Joyson, Professor Zygmunt H. Krukowski, Dr Momin Malik, Department of Surgery, Aberdeen Royal Infirmary; Dr Kirsty McCormack, Mrs Alison McDonald, Mr Graeme MacLennan, Dr Gladys McPherson, Mrs Christiane Pflanz-Sinclair, Professor Craig Ramsay, Health Services Research Unit, University of Aberdeen.

### Writing Committee (alphabetical order)

Mr Irfan Ahmed, Department of Surgery, Aberdeen Royal Infirmary; Dr Jonathan A Cook, Health Services Research Unit, University of Aberdeen; Dr Anne Duncan Health Services Research Unit, University of Aberdeen; Zygmunt H Krukowski, Department of Surgery, Aberdeen Royal Infirmary; Dr Momin Malik, Department of Surgery, Aberdeen Royal Infirmary; Mr Graeme MacLennan, Health Services Research Unit, University of Aberdeen; Dr Kirsty McCormack, Health Services Research Unit, University of Aberdeen.

### Advisory Group

Dr Jennifer Burr, Professor Marion Campbell, Professor Craig Ramsay, Professor John Norrie, Health Services Research Unit, University of Aberdeen.
